# Assessment of the phenotypic severity of hemophilia A: using rotational thromboelastometry (ROTEM) and APTT-clot waveform analysis

**DOI:** 10.1007/s44313-024-00018-6

**Published:** 2024-05-14

**Authors:** Deepika Gupta, Vandana Arya, Jasmita Dass, Nitin Gupta, Manas Kalra, Anupam Sachdeva, Jyoti Kotwal

**Affiliations:** 1 Department of Hematology, Sir Gangaram Hospital, Old Rajinder Nagar, New Delhi, 110060 India; 2 Department of Lab Hematology, All India Institute of Medical Sciences, New Delhi, India; 3 Department of Pediatric Hemato Oncology, Sir Gangaram Hospital, New Delhi, India

**Keywords:** Hemophilia A, ROTEM, APTT-CWA, Phenotype severity, Bleeding disorder, Bethesda assay

## Abstract

**Background:**

Hemophilia A (HA) is an X-linked inherited bleeding disorder caused by reduced factor VIII (FVIII) levels. Approximately 10–15% of patients with severe HA (SHA) do not present with the anticipated bleeding pattern. Here, we assessed the phenotypic severity of hemophilia A using rotational thromboelastometry (ROTEM) and activated partial thromboplastin time-clot waveform analysis (APTT-CWA).

**Methods:**

Patients diagnosed with hemophilia A were enrolled. Clinical phenotype assignment was performed according to the published literature, and patients were classified into four phenotypic subgroups. The whole blood sample was first run on ROTEM in INTEM mode using platelet-poor plasma, APTT was run, and the APTT-CWA graph was simultaneously recorded.

**Results:**

A total of 66 patients were recruited for this study. Statistically significant differences were observed between the four phenotypically categorized groups using ROTEM and APTT-CWA. On comparing patients with mild/moderate-to-severe phenotypes (Group II) with SHA without inhibitors (Group IV), no significant difference was found for all parameters of ROTEM or APTT-CWA. The MCF, MA30, MAXV, and Alpha angle values using ROTEM were found to be the lowest in patients with SHA with inhibitors, which helped differentiate them from those with SHA without inhibitors. However, these two groups could not be differentiated using the APTT-CWA parameters.

**Conclusion:**

ROTEM can be used to distinguish patients with SHA with inhibitors from those with SHA without inhibitors using a combination of parameters with high sensitivity and specificity. However, APTT-CWA cannot be used to differentiate these patient groups.

**Supplementary Information:**

The online version contains supplementary material available at 10.1007/s44313-024-00018-6.

## Introduction

Hemophilia A (HA) is an X-linked inherited bleeding disorder characterized by reduced factor VIII (FVIII) levels [1]. HA is conventionally classified into severe, moderate, and mild categories based on FVIII concentrations of < 1%, 1–5%, and 5–40% respectively. Severely ill patients had frequent spontaneous bleeding unless they received regular prophylactic factor replacement therapy. In contrast, patients with moderate HA experience bleeding at a lower frequency, usually after trauma [2]. It has been observed that 10–15% of patients classified as having severe hemophilia A (SHA) do not present with the anticipated bleeding pattern [2–5]. Similarly, some patients in the moderate category exhibited a severe clinical phenotype with frequent spontaneous bleeding episodes [2–6]. The one-stage activated partial thromboplastin time (APTT)-based FVIII assay is used for the assignment of HA subcategories; however, it has some pitfalls. The assay becomes largely insensitive at values less than 1% and may be affected by factors in the intrinsic pathway of coagulation or by the presence of lupus anticoagulants [7]. The alternative is the two-stage chromogenic FVIII assay, which has a higher precision and sensitivity. However, it is not available in most laboratories that perform coagulation studies worldwide and is expensive [7–9]. Hence, there is a need to supplement the APTT-based FVIII assay, which has led to the exploration of global hemostatic assays such as the thrombin generation test [1], APTT-clot waveform analysis (APTT-CWA) [10], Rotational thromboelastometry [ROTEM] / thromboelastography (TEG) in patients with HA [11, 12].

The routinely reported APTT depends on the action of thrombin on fibrinogen to form fibrin, which does not account for the thrombin generation speed or the total amount of thrombin generated. Modern photo-optical coagulometers are research tools that measure the entire process of clot generation while an APTT is being performed; this curve is referred to as the APTT clot waveform. The velocity of the clotting process and acceleration were monitored as first and second derivatives, respectively [2–4, 13, 14].

Another available assay is ROTEM. This overcomes the in vitro nature of APTT and accounts for a combination of the effects of clotting factors, platelets, leukocytes, and red blood cells on coagulation [5]. ROTEM is useful for monitoring therapy with bypassing agents in patients treated with hemagglutinin and FVIII inhibitors [15]. Although significant work has been performed in the field of hemophilia utilizing APTT, only a few studies have differentiated the clinical phenotypes of hemophilia A [2–4, 7, 10, 16].

Similarly, data and studies are available utilizing TEG in the field of hemophilia A, but there are a very limited number of studies [12, 17] across the world on the phenotypic severity of hemophilia using ROTEM and specifically the INTEM mode. There are differences in the operator characteristics between the ROTEM and TEG; therefore, the results of both are not interchangeable [11]. We studied the utility of APTT-CWA and ROTEM to assess the phenotypic severity in patients with HA.

## Materials and methods

### Patients and study criteria

This study was conducted at the Department of Hematology of a tertiary care center in New Delhi over a period of 16 months, from August 2018 to January 2020. All patients with HA encountered during the study period were enrolled after obtaining written informed consent from the patient or parents/guardians, as applicable. The study was initiated after obtaining permission from the institutional ethics committee. A comprehensive clinical history and clinical examination were recorded to phenotypically categorize the patients into severe and non-severe HA (SHA). For this study, a clinical phenotype assignment was performed as per Shima et al. [18] Newborns (age < 1 year) with spontaneous bleeding, children under 3 years old experiencing initial joint or muscle bleeds, and patients with either intracranial hemorrhage or untreatable oral bleeding were classified as having the SHA phenotype. All other patients were classified as non-SHA [18].

### Blood samples

Peripheral blood samples were collected in two 3mL sodium citrate vacutainers (BD Biosciences, India). One tube was used for ROTEM, and the second tube was used to prepare platelet-poor plasma (PPP). Plasma was separated by centrifugation at 2,000 g (3,500 rpm) for 15 min to prepare the PPP.

### APTT and one-stage factor assay

HA was diagnosed using APTT and a one-stage FVIII assay was performed on the PPP. The PPP was run on ACL TOP750 or ACL TOP700 coagulometer using APTT reagent SynthASil™ (Instrumentation laboratories) and calcium chloride as a re-calcifying agent. Simultaneously, for APTT-CWA, the values were obtained for the 1st and 2nd derivatives. The values of the 1st derivative and 2nd derivative were measured on different coagulometers as min1 and min2 on the MDA and Density Max™ or as peak1 and peak 2 on the ACL TOP analyzers by instrumentation laboratories. The patients were classified as mild, moderate, and SHA according to the value of factor VIII obtained in a one-stage factor assay as 5–40%, 1–5%, and < 1%, respectively.

### ROTEM (INTEM MODE)

The citrated whole blood was subjected to ROTEM using the INTEM mode of analysis within 4 h of sample collection. Samples were automatically pipetted into an instrument using a robotic pipetting procedure. The pipette dispensed 300 μL of citrated whole blood into a plastic cup. The sample was then recalcified, and the test was performed using 20 μL of INTEM reagent containing ellagic acid. All tracings were recorded for up to 1 h. The following clotting parameters were measured: clotting time (CT), clot formation time (CFT), maximum clot firmness (MCF), alpha angle (α), maximum amplitude 30 min after CT (MA30), maximum velocity of clot formation (MAXV), and time to maximum velocity (MAXVT).

### Inhibitor screen and Bethesda assay

All samples with prolonged APTT were subjected to immediate and 2-h incubated APTT inhibitor screens to look for time- and temperature-dependent inhibitors. All patients with a positive inhibitor screen were subjected to Bethesda assay for inhibitor quantification.

### Statistical analysis

All reported continuous variables were expressed as medians and ranges. To compare continuous variables, Mann–Whitney U or Wilcoxon rank-sum tests were performed. Diagnostic accuracy was obtained from receiver operating characteristic (ROC) curve analysis, and the same was performed to determine the optimal cutoffs for the ROTEM and APTT-CWA parameters. All statistical analyses were performed using IBM SPSS Statistics for Windows version 20.

## Results

### Patient characteristics and demographics

A total of 66 patients with HA, including 13 with mild HA, 18 with moderate HA, and 35 with SHA, were enrolled after obtaining written informed consent. All the patients were male. The median age of the study population was 9.5 years (range: 0.5–57 years). The median age at HA diagnosis was 1 year (range: 0.1–36 years). The baseline characteristics of the patients in the three groups are described in Table 1. For the purpose of analysis, mild and moderate HA were clubbed into non-SHA.

**Table 1:** Baseline characteristics of patients with HA according to severity determined by a one-stage FVIII assay

**Character**	**Mild HA (*n* = 13) median (range)**	**Moderate HA (*n* = 18) median (range)**	**Severe HA** (***n* = 35) median (range)**
Age in years (median & range)	21 (5–57)	7 (1–20)	8 (0.5–47)
Age at diagnosis in years (median & range)	5 (0.1–36)	2 (0.1–12)	0.6 (0.2–20)
Bleeding			
Spontaneous/trauma joint bleeding [n (%)]	4 (30.7)	8 (44.4)	34 (97.1)
ICH [n (%)]	1 (7.6)	1 (5.5)	7 (20)
Muscle bleeds [n (%)]	0	3 (16.6)	4 (11.4)
Surgical bleeding [n (%)]	2 (15.3)	2 (11.1)	4 (11.4)
Mucosal bleeding [n (%)]	2 (15.2)	12 (66.6)	30 (85.7)
No bleeding [n (%)]	5 (38.46)	1 (5.5)	0
Factor VIII level (%) Median (range)	21.8 (5.70–29.8)	3.35 (1.0–4.9)	0.4 (0.0–0.9)
Bleeding severity as per Shima et al. [18]			
Severe (n)	2	4	35
Non-severe (n)	11	14	0

To compare APTT-CWA and ROTEM with phenotypic severity, the non-SHA group was further categorized according to phenotypic severity as per Shima et al. [18]. Based on bleeding severity, non-SHA patients were classified into Group I with a non-severe bleeding phenotype comprising 25 patients and Group II with a severe bleeding phenotype comprising 6 patients. In Group I, 11 had mild HA and 14 had moderate HA. In Group II, 2 patients had mild HA and 4 had moderate HA. The SHA patients were further classified into Group III if they had inhibitors and Group IV if they lacked inhibitors. Group III included 14 patients, and Group IV included 21 patients with SHA.

The median ages in Groups I, II, III, and IV were 4 years (0.1–36), 1 year (0.5–12), 0.6 years (0.2–20), and 0.7 years (0.1–3) respectively. The maximum number of patients was observed in the younger age group of 0.1–10 years (54.5%) followed by 11–20 years (27.2%). Similarly, in Groups I and III, 60% and 78.5% were aged 0.5–10 years, respectively. This may be due to the diagnosis at an early age and frequent bleeding in the younger age group, which contributes to frequent hospital visits. In all patients who were on prophylaxis for factor VIII, samples were collected after a washout period of at least 72 h. Most patients received weekly intermediate-to-low-dose prophylaxis. In addition, it was found that out of 14 patients who developed inhibitors, 9 were on prophylaxis for factor VIII.

### APTT-CWA

The variables (1st derivative, 2nd derivative, and max2) of the clot waveform analysis were compared between different groups. CWA parameters were not available in 5 SHA, five with moderate HA, and one with mild HA because of failed APTT results. SHA patients had a significantly shorter 1st derivative, 2nd derivative, and max2 than non-SHA patients (Table 2).

**Table 2:** Distribution of clot waveform parameters between severe and non-severe HA and intergroup comparison between the phenotypic groups

Parameters	Severe HA (SHA) [*N* = 30] median (range)		Non-Severe HA (non-SHA) [*N* = 24] median (range)		*P*-value
1st derivative	38.7 (15.62–134.6)		116.3 (32.62–399.3)		< 0.0001
2nd derivative	42.1 (10–385.2)		227.4 (28.62–1238.5)		< 0.0001
Max2	13.95 (3.12–123.3)		56.0 (7.7–529.8)		< 0.0001
	**Group I** **(n = 25)**	**Group II (n = 6)**	**Group III (n = 14)**	**Group IV (n = 21)**	***P*-value**
**1st derivative**	132.05 (46.7–399.3)	70.45 (32.6–84.9)	36.35 (15.6–70.3)	47.25 (24.4–134.6)	< 0.0001
**2nd derivative**	271.0 (39.6–1238.5)	97.5 (28.62–126.3)	36.75 (10–105.9)	49.05 (16.7–385.2)	< 0.0001
**Max2**	63.3 (7.72–529.8)	30.75 (11.1–58.6)	11.0 (3.1–25)	16.25 (3.8–123.3)	< 0.0001
**GROUPS**	**1st derivative** ***P*-value**		**2nd derivative** ***P*-value**		**MAX2** ***P*-value**
**Group I vs. II**	0.001		0.004		0.025
**Group I vs. III**	< 0.0001		< 0.0001		< 0.0002
**Group I vs. IV**	< 0.0001		< 0.0001		0.001
**Group II vs. III**	0.022		0.022		0.011
**Group II vs. IV**	0.444		0.312		0.274
**Group III vs. IV**	0.108		0.138		0.108

The median ranges for CWA and ROTEM parameters were calculated, and it was found that patients with non-SHA with the non-severe phenotype (Group I) had the maximum velocity and acceleration for clot formation followed by the patients in Group II who were non-SHA but a severe phenotype (Group II). Patients treated with SHA and inhibitors had the lowest velocity and acceleration values in the clot kinetics graphs (Table 2).

The comparison was done for different variables of clot waveform analysis were compared among various phenotypically categorized groups. There was a progressive reduction in all measured parameters from Group I to Group II, and then from Group III to Group IV (Table 2). 1st derivative, 2nd derivative, and max2 differed significantly between Groups I and II, I and III, I and IV, and II and III. It is interesting to note that non-SHA patients with a severe phenotype (Group II) had significant differences from other non-SHA patients (Group I) and SHA patients with inhibitors (Group III), but not with SHA without inhibitors (Group IV). SHA patients with inhibitors (Group III) could not be differentiated from SHA patients without inhibitors using APTT-CWA (Table 2).

Intergroup analysis was performed to derive the cutoff values from the ROC curve analysis for differentiating phenotypically categorized groups. The cutoff values along with the AUC, *P*-value, sensitivity, and specificity for the 1st and 2nd derivatives are shown in Table S1.

In comparison, the 1st and 2nd derivatives were able to differentiate Group II from Groups I and III with statistically significant cut-offs whereas 1st and 2nd derivatives were not able to differentiate Groups II and IV, indicating that the APTT-CWA parameters were similar for non-SHA with severe phenotypes and SHA patients without inhibitors.

This may signify that patients in Group II i.e. non-SHA with severe phenotype behaved like SHA patients without inhibitors in Group IV. Similarly, no statistically significant cutoff was derived to differentiate Groups III and IV.

### ROTEM

Among the INTEM parameters, CT, CFT, and MAX-VT were significantly higher in SHA when compared to the non-SHA group, whereas MCF, alpha angle, MA30, and MAXV were significantly lower (Table 3), indicating relatively better clot formation in patients with mild and moderate HA than in those with SHA.

**Table 3:** Distribution of ROTEM parameters between severe and non-severe HA and intergroup comparison between the phenotypic groups

ROTEM (INTEM)	Severe HA (*n* = 35) median (range)		Non-severe HA (*n* = 31) median (range)		*P*-value
CT	727.0 (251–1852)		343.0 (202–1084)		< 0.0001
CFT	184.0 (69–1904)		76.0 (38–271)		< 0.0001
MCF	64.0 (27–81)		68.0 (47–82)		0.016
ALPHA ANGLE	59.0 (0–76)		75.0 (46–83)		< 0.0001
MA30	64.0 (19–82)		68.0 (46–82)		0.015
MAXV	9.0 (2–37)		17.0 (6–39)		0.00001
MAXVT (seconds)	852.0 (283–1952)		368.0 (252–1299)		0.00001
	Group I (n = 25)	Group II (n = 6)	Group III (n = 14)	Group IV (n = 21)	*P*-value
CT	310.0 (202–1084)	542.0 (360–916)	816.0 (502–1852)	705.0 (251–1141)	< 0.0001
CFT	74.0 (38–271)	120.5 (58–223)	451.0 (122–1904)	137.0 (69–241)	< 0.0001
MCF	68.0 (47–82)	64.5 (52–82)	53.5 (27–81)	66.0 (54–72)	0.019
α ANGLE	75.0 (46–82)	68.0 (51–83)	41.5 (0–71)	64.0 (48–76)	< 0.0001
MA30	68.0 (46–82)	64.0 (52–82)	44.0 (19–81)	67.0 (54–75)	0.0044
MAXV	17 (6.0–39)	14.5 (6.0–37)	5 (2–16)	10 (2.0–20)	< 0.0001
MAX VT	368.0 (252–1299)	645.0 (384–1011)	877.0 (760–1952)	883.0 (282–1339)	< 0.0001

CT was significantly higher in non-SHA with a severe bleeding phenotype than in non-SHA with a non-severe bleeding phenotype but was not significantly different from SHA without inhibitors. This indicated that the bleeding phenotype correlated better with ROTEM than with the one-stage APTT factor VII-based categorization of severity. The CT and CFT were also significantly higher in SHA with inhibitors than in those without inhibitors (Table 3).

**Fig. 1:**
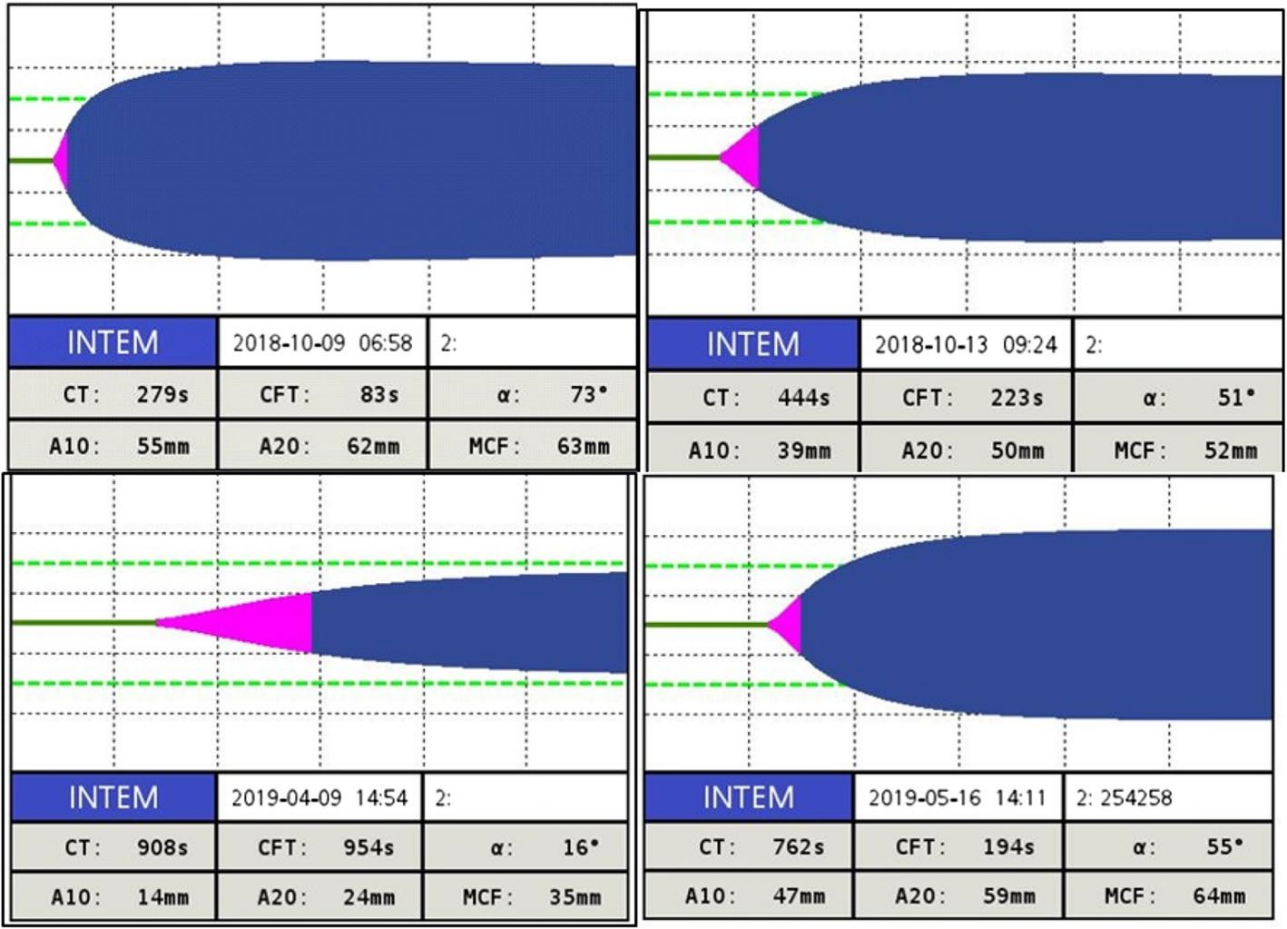
Temograms of the study population in Group I, Group II, Group III, and Group IV

When Group I was compared with Group III, all ROTEM parameters were significantly different. Similarly, the CT, CFT, and alpha angle were significantly different between Groups I and IV, except for MCF and MA30 (Table S2). When comparing Group II with Group IV, no significant difference was found for any of the ROTEM parameters. This can be interrelated, as the group with non-SHA with severe bleeding phenotype behaved similarly to SHA without inhibitors. The temograms of a few selected cases are shown in Fig. 1.

When comparing Groups III and IV, the CFT and alpha angle were significantly different. The CFT was more prolonged among patients with severe hemophilia with inhibitors, and the alpha angle and MCF were decreased in patients with SHA with inhibitors. MA30 and MAXV were also significantly lower in SHA treated with inhibitors (Table S2).

**Fig. 2:**
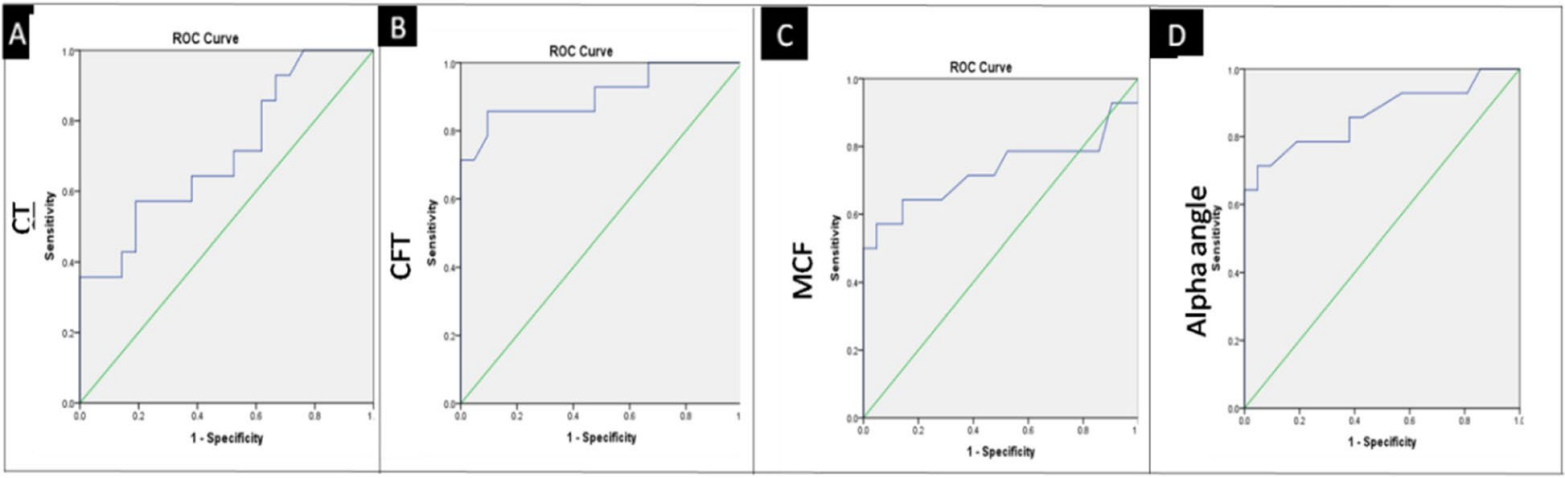
ROC curve analysis to calculate CT, CFT (**A**), MCF, and alpha angle **(B**) value for predicting mild/moderate hemophilia A with severe phenotype when compared to severe hemophilia A with inhibitors with severe phenotype (Group II vs. Group III)

Receiver operating characteristic (ROC) curve analysis was also performed to calculate the optimal cut-off values for the ROTEM parameters (Fig. 2 and S1). All the ROTEM parameters CT, CFT, MCF, MA30, MAXV, and α-angle were able to differentiate SHA without inhibitors (Group III) from SHA with inhibitors (Group IV). Statistically, the best cutoffs were obtained for CFT and alpha angle, followed by MAXV. Table S3 shows the cutoff values for various ROTEM parameters, along with the AUC, p-value, sensitivity, and specificity.

## Discussion

Our study included 66 patients with newly or previously diagnosed HA who were undergoing treatment. We compared the parameters obtained using ROTEM and APTT-CWA between patients with and non-SHA patients.

We found that the maximum coagulation velocity (1st derivative) and maximum coagulation acceleration (2nd derivative) were higher in patients with non-SHA; as the factor level decreased for SHA, the 1st and 2nd derivative values decreased, indicating lower clot kinetics. Upon reviewing the literature, we found that all studies using APTT-CWA were performed on coagulometers that were different from the ones we used. used ACL TOP750 coagulometer. Thus, the values obtained from these studies cannot be compared with ours. Abraham et al*.* [16] from South India carried out similar work on Destiny max™ coagulometer and found lower min1 (coagulation velocity) and min2 (coagulation acceleration) values in SHA as compared to non-SHA. Aghighi et al*.* [19] from the United Kingdom also found similar results using the MDA coagulometer. However, none of these studies specifically addressed patients with SHA and inhibitors. A study from South India [4] on the ACL 10000 (IC, Milan, Italy) assessed the generation of thrombin and clot formation based on photo-optical data and light scatter. This platform is similar to our platform. In their study, FVIII-deficient clinical samples spiked with rFVIII showed a progressive increment of the 2nd derivative on the Clot waveform. This clearly shows that the maximum coagulation acceleration increases as the factor level increases, which is consistent with our results.

We analyzed the differences among the four phenotypically categorized groups. Statistically significant differences were found in the 1st derivative and 2nd derivative values for all four groups (Table 2). In patients with SHA with inhibitors of the severe phenotype (Group III), the median values for 1st and 2nd derivatives were the lowest because of the anticipated absent residual activity of FVIII, followed by Group IV (SHA without inhibitors), and the median values were the highest for non-SHA with the non-severe bleeding phenotype. Abraham et al. [16] reported similar results. We performed intergroup comparisons and found that non-SHA with a non-severe bleeding phenotype (Group I) was statistically different from all other groups with a severe bleeding phenotype. However, we did not find a significant difference between non-SHA patients with a severe bleeding phenotype (Group II) when compared to SHA without inhibitors (Group IV). This may signify that the patients in Group II behaved like patients with SHA without inhibitors. (Table 2). To validate our study, further research is required on the various phenotypes of HA using APTT-CWA on this photo-optic coagulometer model, which is available in numerous laboratories.

We analyzed the ROTEM (INTEM) parameters of the different groups. The advantage of ROTEM over APTT-CWA was that analysis was possible in all 66 cases. CT and CFT in patients with SHA were found to have a median value of 720 s, which is much longer than that in the mild/moderate group. The lower MCF and alpha angle values in the SHA group were suggestive of slow and ineffective clot formation. Statistically significant differences were found in the median values of CT, CFT, MCF, and alpha between groups. Similarly, the maximum velocity of clot formation was reduced in patients with SHA. Furukawa et al. [15] compared severe and moderate hemophilia A and obtained results similar to ours. Similar to our study, Aghighi et al. [19] also studied the role of global assays in measuring the coagulation potential of patients with HA. Except for the few studies quoted above, most of the work using viscoelastic testing in HA is available for TEG.

In our study, we found clinically significant differences in all studied parameters among the different groups based on clinical phenotype. CT and CFT were prolonged in all groups, with the highest being in Group III (SHA with inhibitors), followed by Group IV (SHA without inhibitors), and non-SHA with a bleeding phenotype. The CT values were the lowest in the non-severe phenotype group and the highest in the severe phenotype group with inhibitors. The MCF and alpha angle were highest for Group I, depicting the highest clot strength in non-SHA patients who behaved non-severe phenotypically, whereas they were lowest for patients with SHA with inhibitors. MA30 was the lowest among the SHA treated with inhibitors. The maximum velocity (MAXV) of clot formation was highest in non-SHA patients and lowest in patients with the SHA phenotype. Similarly, the time to achieve maximum velocity (MAXVT) was much longer in patients with SHA, indicating ineffective clot kinetics.

FVIII inhibitors develop in approximately 30% of patients with severe or moderate SHA in response to the infusion of FVIII replacement therapy. FVIII inhibitors are typically detected using the Bethesda, Nijmegen Bethesda, or enzyme-linked immunosorbent assays [16]. However, these tests are generally unavailable in routine clinical laboratories at local hospitals. In our study, the MCF, MA30, MAXV, and Alpha angle values were lowest in patients with SHA treated with inhibitors. These parameters can be utilized as screening tools in patients receiving prophylactic therapy and requiring testing for inhibitors. In a resource-poor country where tests such as the Bethesda assay are technically challenging and not widely available, these ROTEM parameters can be utilized as a screening tool for these patients to look for the development of inhibitors. In many studies, ROTEM has been used to guide treatment with passing agents in patients with hemophilia and inhibitors. In a study by Chitlur et al. [12], similar results were reported on TEG. All evaluated ROTEM parameters could differentiate Group III from Group IV very well. A combination of all parameters would make the test highly sensitive and specific for distinguishing between the two groups. Thus, ROTEM plays a major role as point-of-care equipment in SHA to distinguish cases with inhibitors from those without inhibitors and has value as a screening test.

Group II, which comprised the mild/moderate patients, contained few individuals exhibiting severe phenotypes. We also identified no patients with SHA displaying a non-severe phenotype in this group. Another limitation is that a chromogenic assay was not performed because of its limited availability throughout the country. Because it is more sensitive, we may have missed a few patients with moderate SHA with a one-stage assay. In addition, molecular studies could not be performed because of their limited availability.

## Conclusions

Using the first and second derivatives, we found that APTT-CWA could distinguish non-SHA patients with severe phenotypes from those with non-severe phenotypes using the 1st derivative and 2nd derivative. Similarly, ROTEM, a point-of-care equipment, can distinguish between the two groups. Among all the parameters analyzed, CFT was the best to differentiate Severe HA with inhibitors from SHA without inhibitors followed by α-angle. In a resource-poor country, where tests to detect inhibitors in patients with HA, such as the Bethesda assay, are technically challenging and not widely available, we found that ROTEM can be used to distinguish SHA patients with inhibitors from SHA patients without inhibitors using a combination of parameters with high sensitivity and specificity. However, APTT-CWA could not differentiate between the two groups. Further research is warranted to enable us to understand the variability in clinical manifestations. Larger prospective studies are required to further evaluate these parameters.

### Supplementary Information

**Supplementary Material 1.**

## Data Availability

All data generated or analysed during this study are included in this published article.
