# Supplementary material for: Assessment of the phenotypic severity of hemophilia A: using rotational thromboelastometry (ROTEM) and APTT-clot waveform analysis
Source: Blood Res. 2024 May 14;59(1):19. doi: 10.1007/s44313-024-00018-6 (PMC11093952; doi:10.1007/s44313-024-00018-6)
Supplement: Supplementary file 1 — Supplementary Material 1. [file 44313_2024_18_MOESM1_ESM.docx]

**Table S1 Distribution of clot waveform parameters between patients with severe and mild/moderate HA**

| **Parameters** | **Severe HA**  **(n=20)** | **Mild/moderate HA (n=30)** | ***P*-value** |
| --- | --- | --- | --- |
| **APTT-clot waveform** | | | |
| **1 ^st^ derivative** | 38.750 (15.62-134.6) | 116.35 (32.62-399.3) | <0.0001 |
| **2^nd^ derivative** | 42.10 (10-385.2) | 227.45 (28.62-1238.5) | <0.0001 |
| **Max2** | 13.95 (3.12-123.3) | 56.0 (7.7-529.8) | <0.0001 |
|  | **Severe HA**  **(n=35)** | **Mild/moderate HA (n=31)** | ***P*-value** |
| **ROTEM** | | | |
| **CT** | 727.0 (251-1852) | 343.0 (202-1084) | <0.0001 |
| **CFT** | 184.0 (69-1904) | 76.0 (38-271) | <0.0001 |
| **MCF** | 64.0 (27-81) | 68.0 (47-82) | 0.016 |
| **α ANGLE** | 59.0 (0-76) | 75.0 (46-83) | <0.0001 |
| **MA30** | 64.0 (19-81) | 68.0 (46-82) | 0.01 |
| **MAXV** | 9.0 (2.0-20) | 17.0 (6-39) | <0.0001 |
| **MAXVT** | 883.0 (283-1952) | 507.0 (355-1096) | <0.0001 |

**Table S2 Group-wise comparisons of APTT-clot waveform parameters**

| **GROUPS** | **1^st^ derivative**  ***P*-value** | **2^nd^ derivative**  ***P*-value** | **MAX2**  ***P*-value** |
| --- | --- | --- | --- |
| **Group I vs. II** | 0.001 | 0.004 | 0.025 |
| **Group I vs. III** | <0.0001 | <0.0001 | <0.0002 |
| **Group I vs. IV** | <0.0001 | <0.0001 | 0.001 |
| **Group II vs. III** | 0.022 | 0.022 | 0.011 |
| **Group II vs. IV** | 0.444 | 0.312 | 0.274 |
| **Group III vs. IV** | 0.108 | 0.138 | 0.108 |

**Table S3 Group-wise comparison of ROTEM parameters**

| **Groups/*P*-value** | **CT** | **CFT** | **MCF** | **ALPHA ANGLE** | **MA30** | **MAXV** | **MAXVT** |
| --- | --- | --- | --- | --- | --- | --- | --- |
| Group I vs. II | 0.015 | 0.208 | 0.679 | 0.608 | 0.51 | 0.74 | 0.91 |
| Group I vs. III | <0.0001 | <0.0001 | 0.004 | <0.0001 | 0.0011 | <0.0001 | <0.0001 |
| Group I vs. IV | <0.0001 | <0.0001 | 0.114 | <0.0001 | 0.20 | <0.0001 | <0.0001 |
| Group II vs. III | 0.091 | 0.003 | 0.109 | 0.006 | 0.07 | 0.0083 | 0.200 |
| Group II vs. IV | 0.629 | 0.408 | 0.977 | 0.408 | 0.74 | 0.24 | 0.39 |
| Group III vs. IV | 0.037 | <0.0001 | 0.022 | <0.0001 | 0.004 | 0.002 | 0.42 |
